# Effect of clinical decision support systems on clinical outcome for acute kidney injury: a systematic review and meta-analysis

**DOI:** 10.1186/s12882-021-02459-y

**Published:** 2021-08-04

**Authors:** Youlu Zhao, Xizi Zheng, Jinwei Wang, Damin Xu, Shuangling Li, Jicheng Lv, Li Yang

**Affiliations:** 1 Renal Division, Peking University First Hospital, Peking University Institute of Nephrology; Key Laboratory of Renal Disease, Ministry of Health of China; Key Laboratory of CKD Prevention and Treatment, Ministry of Education of China; Research Units of Diagnosis and Treatment of Immune-mediated Kidney Diseases, Chinese Academy of Medical Sciences, 8 Xishiku ST, Xicheng District, 100034 Beijing, People’s Republic of China; 2 Surgical Intensive Care Unit, Peking University First Hospital, Beijing, China

**Keywords:** Acute kidney injury, Care bundle, Electronic alert, Clinical decision support system

## Abstract

**Background:**

Clinical decision support systems including both electronic alerts and care bundles have been developed for hospitalized patients with acute kidney injury.

**Methods:**

Electronic databases were searched for randomized, before-after and cohort studies that implemented a clinical decision support system for hospitalized patients with acute kidney injury between 1990 and 2019. The studies must describe their impact on care processes, patient-related outcomes, or hospital length of stay. The clinical decision support system included both electronic alerts and care bundles.

**Results:**

We identified seven studies involving 32,846 participants. Clinical decision support system implementation significantly reduced mortality (OR 0.86; 95 % CI, 0.75–0.99; *p* = 0.040, I^2^ = 65.3 %; *n* = 5 studies; *N* = 30,791 participants) and increased the proportion of acute kidney injury recognition (OR 3.12; 95 % CI, 2.37–4.10; *p* < 0.001, I^2^ = 77.1 %; *n* = 2 studies; *N* = 25,121 participants), and investigations (OR 3.07; 95 % CI, 2.91–3.24; *p* < 0.001, I^2^ = 0.0 %; *n* = 2 studies; *N* = 25,121 participants).

**Conclusions:**

Nonrandomized controlled trials of clinical decision support systems for acute kidney injury have yielded evidence of improved patient-centered outcomes and care processes. This review is limited by the low number of randomized trials and the relatively short follow-up period.

**Supplementary Information:**

The online version contains supplementary material available at 10.1186/s12882-021-02459-y.

## Background

As a common disorder occurring in up to 22 % of hospitalized patients [1], and more than 50 % of the critically ill [2], acute kidney injury (AKI) is associated with high in-hospital mortality rates (> 20 %) [1], prolonged hospital stays and increased healthcare expenditure. Inadequate detection and management were closely related to the poor outcomes of AKI patients [3–5] and were highlighted as a challenge for healthcare systems, particularly in developing countries [6]. In a nationwide, cross-sectional survey of AKI in China [7], the nonrecognition rate of AKI was up to 74.2 %, and 17.6 % of patients with recognized AKI were given a delayed diagnosis, which was further shown to be an independent risk factor for in-hospital death.

As recommended by the Acute Dialysis Quality Initiative (ADQI) consensus [8], information technology is increasingly being used in the healthcare setting worldwide to automatically recognize AKI and send electronic alerts (e-alerts) to physicians. However, except for effectively changing physician behavior, e-alerts alone did not improve the clinical outcomes of AKI patients [9–12]. Therefore, care bundles were implemented in conjunction with e-alerts to construct integrated clinical decision support system. The system has been widely implemented in sepsis, mechanical ventilation and central venous catheters usage, with promoted compliance in process of care variables and beneficial clinical outcomes. The system effectively reduced catheter-related bloodstream infection and ventilator-associated pneumonia [13–15]. Care bundles are defined as a structured set of straight forward and evidence-based practices, treatments and interventions designed to improve the processes of care delivery and ultimately outcomes. Potential elements of AKI care bundles design could be monitoring kidney function, assessment for drugs with renal toxicity, volume assessment and so on [16]. There is a strong rationale for their use in AKI, and the evidence base around clinical decision support system is growing but conflicting. Existing data suggested that the process of care could be improved in various degrees [17, 18]. However, the impact on patient’s overall and renal outcomes are inconsistent [18, 19].

Hereby, we systematically review the studies that evaluate the effect of e-alerts and care bundles on the clinical outcomes of patients with AKI. We focused mainly on the characteristics of AKI alerting systems and care bundle contents, especially the effects of this system on clinical outcomes.

## Methods

### Search strategy and selection criteria

We performed a systematic review and meta-analysis adhering to the statement for the conduct of meta-analyses of intervention studies and Preferred Reporting Items for Systematic Reviews and Meta-Analyses (PRISMA) (Additional file 1: Appendix 1). The protocol was registered in the International Prospective Register of Systematic Reviews (PROSPERO; Identifier CRD42020163856). We included original research articles including randomized, before-after, and cohort studies of hospitalized patients with both AKI e-alerts and care bundles implemented. Studies must have clear definitions for AKI, and describe its impact on care processes, patient-centered outcomes, or hospital length of stay. Studies with either e-alerts or care bundles alone were excluded.

Relevant studies were identified by searching Medline (from 1990 to 2019), Embase (from 1990 to 2019), and the Cochrane Library database (Cochrane Central Register of Controlled Trials; no date restriction) were searched. The search strategy was developed in consultation with an expert research librarian (GH R) (Additional file 1: Appendix 2). The literature search, data extraction, and quality assessment were undertaken independently by two authors (YL Z, XZ Z). Disagreements were resolved through discussion with a third reviewer (L Y).

### Data extraction

Relevant information was extracted into a standardized spreadsheet, which included sample size, baseline patient characteristics [age, sex, history of chronic kidney disease (CKD)], clinical course and prognosis of AKI, follow-up duration, clinical decision support system especially care bundles details, care processes, outcome events (patient-related outcomes, length of stay). We assessed methodological quality for randomized controlled trials (RCTs) using the Cochrane collaboration’s tool for assessing risk of bias [20]. Other non-randomized experimental studies were assessed by methodological index for non-randomized studies (MINORS), a validated tool to discern the methodological quality of nonrandomized studies [21].

The process of care included medication reviews (defined by nephrotoxin dose adjustment or discontinuation, or medical chart review), AKI recognition (defined by documented AKI in clinical notes), fluid assessment, and investigations (defined by approaches looking for the major causes of AKI or monitoring renal function, which include urinalysis, serum creatinine, other laboratory examinations, and kidney ultrasonography). Because the process of care lacking comparisons between the intervention and control groups, missing data, or heterogeneity among studies, only AKI recognition and investigations were eventually incorporated in the meta-analysis. Other management practices, including care bundle usage, nephrology consultation, and risk assessment, were presented solely in tables.

### Outcomes

The primary outcome was all-cause mortality. Secondary outcomes were receipt of renal replacement therapy (RRT), AKI progression (defined as an increase in AKI stages), and hospital length of stay. Other renal outcomes included renal recovery and AKI duration.

### Statistical analyses

We obtained summary estimated of odds ratios (ORs) with 95 % confidence intervals (CIs) for categorical variables and weighted standard mean differences with 95 % CIs for continuous variables by using a random-effects or fixed-effects model. Statistical heterogeneity was assessed using I^2^ statistic. The sensitivity analysis was conducted to test if a particular study contributed appreciably to the observed heterogeneity by excluding studies with extreme ORs from the meta-analysis. Publication bias was tested by the Egger’s test. Subgroup analysis by mortality observed at different times was performed. A two-sided p value less than 0.05 was regarded as statistically significant. Study quality graph was presented with Review Manager (RevMan version 5.3.5, Copenhagen: The Nordic Cochrane Centre, The Cochrane Collaboration, 2014). Meta analyses were performed using STATA (version 15; Stata Corp, College Station, TX, USA).

## Results

### Study selection

**Fig. 1:**
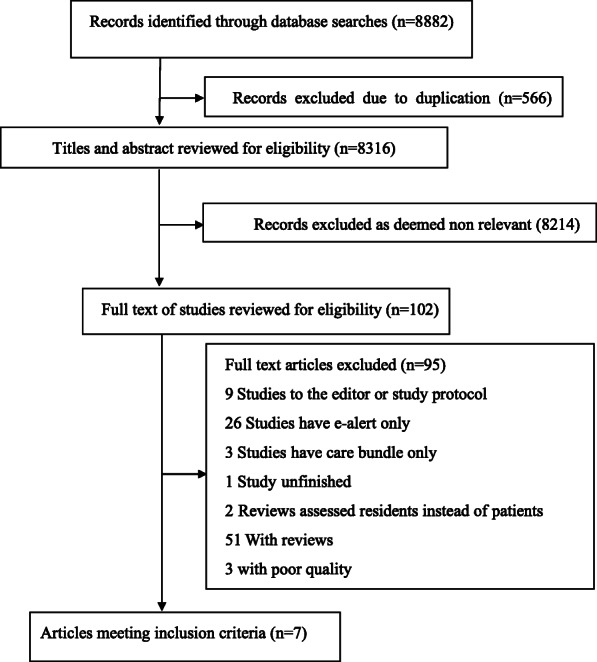
Flow chart for study inclusion

Our search yielded 8882 articles (Fig. 1**)**. Of 102 articles reviewed in full text, seven studies met the eligibility requirements [17–19, 22–25]. Of the included studies, two were RCTs [17, 18], three were before-after designs [23–25], one used a propensity score-matched cohort [19] and the other one was a prospective cohort [22] **(**Table 1**).**

**Table 1:** Characteristics of included studies

Study	Design	Country	Setting	Number of patients or AKI events
Hodgson 2018 [23]	Controlled before-after	UK	Mix	1062
Kolhe 2015 [22]	Prospective cohort	UK	Mix	2500
Kolhe 2016 [19]	Propensity score-matched cohort	UK	Mix	2762
McCoy 2010 [24]	Before-after	USA	Mix	1659
McCoy 2012 [17]	Randomized controlled trial	USA	Mix	396
Selby 2019 [18]	Randomized controlled trial	UK	Mix	24,059
Thomas 2014 [25]	Before-after	UK	Mix	408

### Study characteristics

**Fig. 2:**
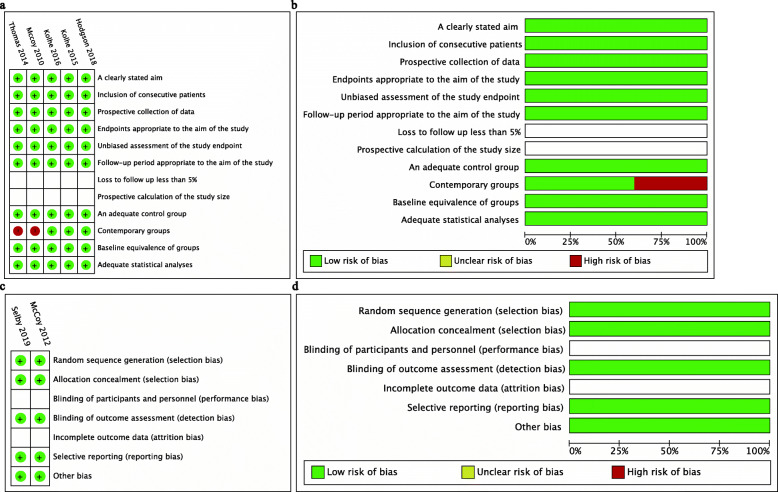
Risk of bias summary of included studies. **a** Risk of bias details of each study by methodological index for non-randomized studies (MINORS); **b** Risk of bias summary by MINORS; **c** Risk of bias details of each study by Cochrane collaboration’s tool; **d** Risk of bias details by Cochrane collaboration’s tool

Table 2 summarizes the characteristics of patients with AKI. The mean age was 74.2 years (interquartile range, 59.5–76.3). Male patients accounted for 48.2 % of the sample. The incidence of AKI was approximately 7.58 %, and more than half of the patients had a moderate to severe stage of AKI. Patients suffering from AKI progression were reported to account for 6.02 % of the sample. RRT support accounted for 2.66 % of involved patients. The average length of stay was 11.9 days (interquartile range, 10.1-16.3). The AKI population was subject to substantial in-hospital mortality or follow-up mortality (22.74 and 27.07 %, respectively). *Risk of bias and quality of evidence.* All studies had moderate to good quality of reporting **(**Fig. 2).

**Table 2:** Characteristics of included participants

Study	Mean follow-up,days	Mean age,years	Male,%	CKD,%	AKI						Mean los,days	Mortality	
					Incidence,%	Etiology	Stages,%	Progression,%	RRT,%	Duration,days		In-patient,%	Follow-up,%
Hodgson2018 [23]	Discharge	74.2	NA	NA	7.2	NA	NA	6.1	NA	NA	14.4	23.0	NA
Kolhe2015 [22]	134	76.9	50.0	NA	NA	Pre renal 71.9 %, renal 9.8 %, post renal 4.5 %	Stage-1 54.1 Stage-2 25.1 Stage-3 20.8	6.3	NA	NA	11.9	22.4	30.2
Kolhe2016 [19]	171	76.3	49.0	NA	NA	Pre renal 56.8 %, renal 11.1 %, post renal 8.1 %	Stage-1 51.3 Stage-2 26.4 Stage-3 22.3	5.7	2.9	NA	11.2	22.9	41.8
McCoy2010 [24]	NA	59.5	56.2	NA	NA	NA	NA	NA	NA	NA	NA	NA	NA
McCoy2012 [17]	NA	59.5	56.8	NA	NA	NA	NA	NA	NA	NA	NA	NA	NA
Selby2019 [18]	30	76.0	49.4	22.6	7.6	NA	Stage-1 62.22 Stage-2 20.7 Stage-3 17.1	NA	5.3	NA	9.0	NA	24.5
Thomas2014 [25]	1460	70.6	46.6	NA	NA	NA	Stage-1 36.0 Stage-2 37.3 Stage-3 26.7	NA	7.9	NA	18.1	NA	59.6

Note: Abbreviations: *AKI* Acute kidney injury, *CKD* Chronic kidney disease, *NA* Not available, *IQR* Interquartile range, *RRT *Renal replacement therapy, *los* length of stay

### Clinical decision support system

#### Delivery methods

In all the enrolled studies, e-alerts were generated and delivered to physicians through electronic medical record (EMR). In Selby’s study, an additional phone call would be made by the duty biochemist to the attending physician if the patient was classified as moderate-severe AKI (AKI stage 2–3) [18]. The contents of care bundles were ummarized in Table 3.

#### Risk assessment

Only Hodgson et al. assessed the risk of developing AKI [23]. By combining multiple predictors such as medical history, age and physiological parameters to calculate an AKI-Predict-Score, his study stratified patients at different risks of AKI according to the AKI-Predict-Score and further provided corresponding therapy. 

#### Medications

The process measure improvements of all studies included medication reviews. All the included studies recommended the review of drug charts and adjustment for potential nephrotoxicity, including contrast media, angiotensin-converting enzyme inhibitors, angiotensin receptor blockers, non-steroidal anti-inflammatory drugs and diuretics [17–19, 22–25]. The McCoy team [17, 24] provided detailed recommendations to modify or discontinue specific drugs. 

#### Nephrology consult

Kolhe [19, 22] and Selby et al [18] recommended a nephrology consult for patients in AKI stage 3 and potential specific causes for AKI. In Thomas’s study, a nephrology consultation was requested, and overall management for AKI was performed [25]. In Hodgson's study [23], a discussion with nephrology is recommended if the patient's condition did not improve.

#### Fluid assessment

Most of the studies highlighted the importance of fluid balance and volume assessment, including urine output measurement [18, 19, 22, 23, 25]. 

#### Investigation

Laboratory tests that help search for underlying causes of AKI, including urinalysis, renal ultrasound, X-rays and other specific blood tests for patients, are generally requested [18, 19, 22, 23, 25].

**Table 3:** Summary of care bundles contents

Study	AKI risk assessment	Medications	Nephrology	Fluid assessment	Investigation
Hodgson 2018 [23]	Yes	Yes	Yes	Yes	Yes
Kolhe 2015 [22]	No	Yes	Yes	Yes	Yes
Kolhe 2016 [19]	No	Yes	Yes	Yes	Yes
McCoy 2010 [24]	No	Yes	No	No	No
McCoy 2012 [17]	No	Yes	No	No	No
Selby 2019 [18]	No	Yes	Yes	Yes	Yes
Thomas 2014 [25]	No	Yes	Yes	Yes	Yes

#### Other aspects

Other aspects that support the management of AKI are also suggested, for instance, treatment of the underlying causes, nutritional assessment, physiotherapy, care pathways, escalation and palliative care [18, 19, 22, 23, 25].

### Clinical outcome

Clinical outcome was summarized in Table 4.

**Table 4:** Summary of clinical outcomes and care process assessment of included studies

Study	Mortality	Receipt of RRT	AKI Progression	AKI duration	AKI recovery	Los	Process of care					
							Care bundle usage	Medication review	Nephrology consult	AKI recognition	Fluid assessment	Investigation
Hodgson2018[23]	Yes	No	Yes	No	No	Yes	Yes	Yes	No	Yes	Yes	Yes
Kolhe2015[22]	Yes	No	Yes	No	No	Yes	Yes	No	Yes	Yes	Yes	Yes
Kolhe2016[19]	Yes	Yes	Yes	No	No	Yes	Yes	No	Yes	Yes	Yes	Yes
McCoy2010[24]	No	No	No	No	No	No	Yes	Yes	No	No	No	No
McCoy2012[17]	No	No	No	No	No	No	Yes	Yes	No	No	No	No
Selby2019[18]	Yes	Yes	Yes	Yes	No	Yes	Yes	Yes	Yes	Yes	Yes	Yes
Thomas2014[25]	Yes	Yes	No	No	No	Yes	No	Yes	Yes	No	Yes	Yes

Note: Abbreviations: *AKI* Acute kidney injury, *RRT* Renal replacement therapy, *Los* length of stay

#### Mortality

**Fig. 3:**
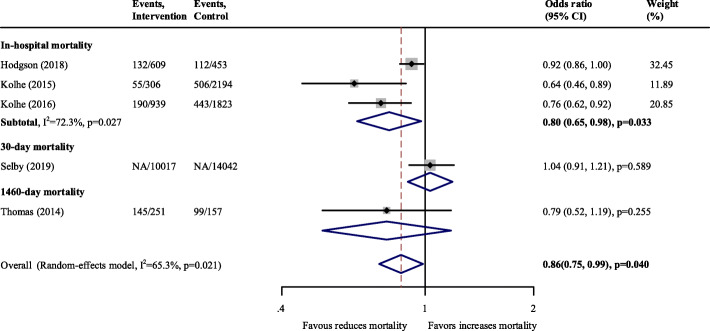
Pooled effect estimates for the impact on mortality

In the pooled analysis, clinical decision support system implementation showed a reduction in overall mortality (OR 0.86; 95 % CI, 0.75–0.99; *p* = 0.040; *n* = 5 studies; *N* = 30,791 participants; I^2^ = 65.3 %) (Fig. 3). In the subgroup analysis according to mortality observed at different time points, there was a decrease in in-hospital mortality (OR 0.80; 95 % CI, 0.65–0.98; *p* = 0.033; *n* = 3 studies; *N* = 6324 participants; I^2^ = 72.3 %), whereas the trend was not favorable regarding follow-up mortality (Fig. 3). No significant publication bias can be seen on the Egger’s test (*P* = 0.283, Supplementary Fig. 1).

#### Length of stay

**Fig. 4:**
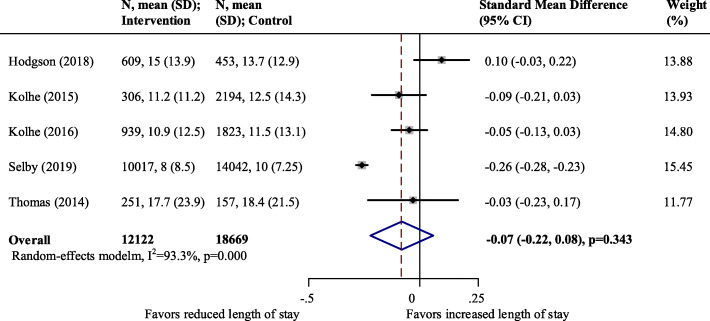
Pooled effect estimates for the impact on length of stay

Five studies (*N* = 30,791 participants) reported hospitalization days, where clinical decision support system performance was not associated with a reduced length of stay [standard mean difference − 0.07; 95 % CI (-0.22- 0.08); *p* = 0.343; I^2^ = 93.3 %] (Fig. 4). To assess the influence of individual studies on the pooled result, we conducted a sensitivity analysis by omitting one study in each turn (supplementary Fig. 2). The I^2^ dropped to 44.0 % after removing the study by Selby [18], with a materially unchanged result [standard mean difference − 0.02, 95 %CI (-0.10 − 0.06), *p* = 0.612] (the forrest plot seen in the supplementary Fig. 3). Publication bias can be seen on the Egger’s test (*p* = 0.042, Supplementary Fig. 4).

#### Renal outcome

**Fig. 5:**
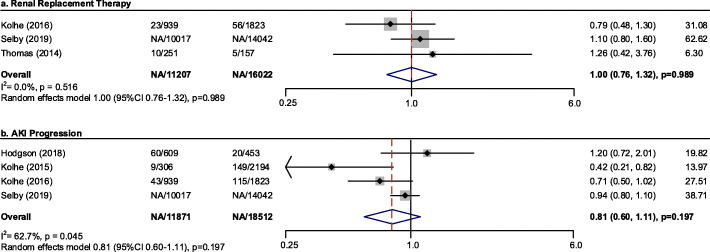
Pooled effect estimates for the impact on renal outcome

In a meta-analysis of three studies (27,229 participants), there was no difference observed in RRT usage between groups with or without intervention (OR, 1.002; 95 % CI, 0.76 to 1.32; I^2^ = 0.0 %) (Fig. 5a). Clinical decision support system usage was nonsignificantly associated with lower odds of AKI progression (OR, 0.81; 95 % CI, 0.60 to 1.11; I^2^ = 62.7 %, *n* = 4 studies, *N* = 30,383 participants) (Fig. 5b). Sensitivity analysis was conducted by omitting one study in each turn (supplementary Fig. 5). The I^2^ dropped to 36.0 % after removing the study by Kolhe [22], with a materially unchanged result (OR, 0.91; 95 %CI 0.73–1.13, *p* = 0.382) (the forrest plot seen in the supplementary Fig. 6). No significant publication bias can be seen on the Egger’s test regarding the above two renal outcomes (*p* = 0.995 and *p* = 0.469, respectively, Supplementary Figs. 7–8).

#### Process of care

**Fig. 6:**
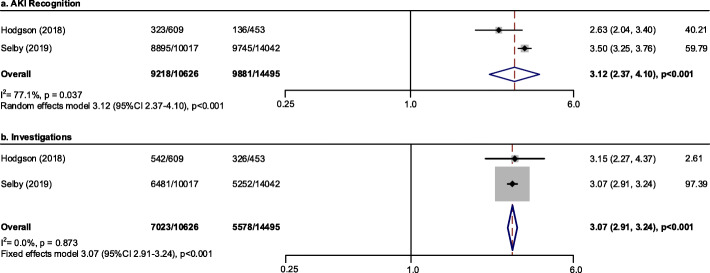
Pooled effect estimates for the impact on care process

Heterogeneity was observed in terms of different processes of care. Adherence to a clinical decision support system was associated with higher odds of AKI recognition (OR 3.12; 95 % CI, 2.37–4.10; *p* < 0.001; *n* = 2 studies; *N* = 25,121 participants; I^2^ = 77.1 %) (Fig. 6a) and investigations (OR 3.07; 95 % CI, 2.91-3.24; *p *< 0.001; *n* = 2 studies; *N* = 25,121 participants; I^2^ = 0.0 %) (Fig. 6b). As studies showed significant heterogeneity with regards to medications review and fluid assessment, meta-analysis was not conducted regarding these two processes of care.

## Discussion

 This systematic review and meta-analysis described the characteristics of clinical decision support system implementation and evaluated its effects on clinical outcome. The study found that clinical decision support system varied in design. Care bundles mostly focus on AKI risk assessment, medication review and adjustment, nephrology consultation, fluid assessment and completion of investigations. In the pooled analysis, the combined intervention significantly improved the clinical outcomes of patients with AKI, including reducing all deaths or in-hospital death events. It also improved AKI recognition and corresponding investigations. System usage did not shorten hospital length of stay and reduce the risk of AKI progression or RRT usage.

This overview systemically evaluated the effects of e-alerts coupled with care bundles and found a clear benefit on the clinical outcomes of patients with AKI. However, e-alerts alone did not improve these outcomes of AKI, including mortality [9, 11, 12], kidney function, AKI progression [11] and frequency of RRT usage [12], as well as health service use comprising hospital stay [11], intensive care unit duration [12], and total pharmacy or hospital costs [9, 11]. In most studies with e-alerts alone, physician behavior improved, such as a shorter time to modification of medications [11]; more reasonable application of fluid therapy, diuretics or vasopressors [12]; or earlier detection of AKI [21] (Supplementary Table 1, [9, 11, 12, 26–34]). Only one study in which e-alerts automatically engaged nephrology teams when AKI occurred showed that e-alerts considerably reduced the rate of severe AKI and promoted AKI recovery [27]. Another three studies including care bundles only (Supplementary Table 2, [35–37]) suggest that care bundles can potentially improve the process of care, including AKI recognition, care bundle completion [35], fluid status assessment, and appropriate investigation and cessation of medications contributing to AKI [36, 37]. These findings, as well as our overview, highlight the importance of e-alerts coupled with care bundles for AKI intervention.

Until now, few trials have evaluated the effects of e-alerts coupled with care bundles. One enrolled a small sample size and focused on pharmacy surveillance-related management without reporting patient-centered or medical resource-associated outcomes [17]. Another RCT [18] was a stepped wedge cluster randomized trial, which failed to show benefits on the primary outcome of mortality but significantly reduced the duration of AKI and the length of hospitalization. In spite of robust methodology, incomplete intervention coverage across participating sites might affect the result. In addition, mortality associated with AKI is a complex condition with multiple etiologies or, rather, a kaleidoscope of comorbidities and coexisting acute illness; therefore, it is not the only indicator of primary outcomes. Moreover, the study did provide evidence that a combined intervention can bring benefits such as reductions in both length of hospitalization and duration of AKI, which are also meaningful positive effects of these combined interventions.

This meta-analysis revealed a decrease in in-hospital mortality; nevertheless, the trend was not favorable regarding long-term mortality. Although pooled together, the overall mortality was lowered, and the included studies had a relatively short follow-up period. Long-term mortality associated with AKI is driven by multiple factors, including the effects of comorbidity and coexisting acute illness [18]. In a large cohort study of patients who initially survived hospitalization with AKI, 28 % of patients died in the subsequent year after discharge, and the most common causes of death were cardiovascular disease (28 %) and cancer (28 %) [38]. CKD may lower the threshold for developing AKI. However, the percentage of preexisting CKD is lacking in the included study, where only one study reported that CKD was present in 22.6 % of the enrolled participants (Table 2). Furthermore, even mild renal dysfunction may predispose patients to CKD, and thus, it increases the risk of subsequent AKI recurrence and ultimately end stage renal disease [39]. In a systematic review of 19 cohort studies, the prevalence of CKD over the next three years after hospital discharge was nearly threefold higher among patients with AKI than among those without AKI [40]. Moreover, a recent large registry study demonstrated an association between CKD and death [41]. However, the included studies rarely reported renal recovery. Our study discovered that the intervention was nonsignificantly associated with lower odds of AKI progression, as once AKI progressed to stage 3 or initiated RRT, it became difficult to halt progression and escalation, which highlighted the importance of the completion of early intervention. In this study, there was no difference observed in RRT usage between groups with or without a clinical decision support system. Possibly due to the reason that patients who undergo RRT have the most severe form of AKI, and the addition of RRT to the ongoing support of critically ill patients would contribute to an increase in complexity and expenditure [26]. Therefore the benefit for starting a critically ill patient on RRT would have to be balanced between addition to bedside workload or resource utilization, and impact on patient’s and family’s preferences for care [42].

This study is the first systematic review and meta-analysis on the effect of e-alerts in combination with care bundles on outcomes. The study included a large sample size and noted a beneficial outcome. However, several limitations are present. First, there was a lack of RCTs identified and included. Second, the follow-up period among the included studies was relatively short. Only one study [25] with a longer-term follow-up of approximately four years found that a nonsignificantly improved survival appeared immediately, consistent with an effect due to the intervention, yet then attenuated further. Besides, due to the scarcity of data on renal outcomes including RRT usage, progression, duration and recovery of AKI, meta-analysis could not be conducted on the AKI recovery or duration. And this would lead to a result coincided with the findings of the limited studies that had been included. What is more, heterogeneity exits in “the process of care” among different studies, especially in “medication reviews” and “fluid assessment”, which was partly related to difference in definitions and assessment methods. Therefore, meta-analysis was not conducted to pool results regarding these two care processes. Finally, as all the included studies were conducted in either the United Kingdom or the United States, the results may not be generalizable to other populations.

## Conclusions

In conclusion, this systematic review and meta-analysis indicates that the combined implementation of e-alerts and care bundles showed a reduction in overall mortality and in-hospital mortality. It also promoted process of care containing AKI detection and relevant investigations. Studies with e-alerts only could help improve AKI recognition and process of care. When combined with treatment recommendations, clinical decision support system employment could ameliorate short-term clinical outcomes. However, RCTs with long-term follow-up conducted in clinical practice in the near future are imperative.

## Supplementary Information

**Additional file 1: Appendix 1.**  PRISMA 2009 Checklist. **Appendix 2.** Search strategy. **Supplementary Figure 1.** The Egger’s test of mortality. **Supplementary Figure 2.** Sensitivity analysis of length of stay. **Supplementary Figure 3.** Pooled effect estimates for the impact on length of stay after removing the study by Selby [18]. **Supplementary Figure 4.** The Egger’s test of length of stay. **Supplementary Figure 5.** Sensitivity analysis for AKI progression. **Supplementary Figure 6.** Pooled effect estimates for the impact on AKI progression after removing the study by Kolhe [22]. **Supplementary Figure 7.** The Egger’s test of AKI-RRT usage. **Supplementary Figure 8.** The Egger’s test of AKI progression. **Supplementary Table 1.** Summary of clinical outcome for studies using only e-alert system. **Supplementary Table 2.** Studies with a design of care bundles only.

## Data Availability

Not applicable.

## Abbreviations

AKI: Acute kidney injury
ADQI: Acute Dialysis Quality Initiative
E-alerts: Electronic alerts
PRISMA: Preferred Reporting Items for Systematic Reviews and Meta-Analyses
PROSPERO: Prospective Register of Systematic Reviews
CKD: Chronic kidney disease
RCTs: Randomized controlled trials
MINORS: Methodological index for non-randomized studies
RRT: Renal replacement therapy
ORs: Odds ratios
CIs: Confidence intervals
EMR: Electronic medical record
