# Supplementary material for: Effect of clinical decision support systems on clinical outcome for acute kidney injury: a systematic review and meta-analysis
Source: BMC Nephrol. 2021 Aug 4;22:271. doi: 10.1186/s12882-021-02459-y (PMC8335454; doi:10.1186/s12882-021-02459-y)
Supplement: Supplementary file 1 — Additional file 1: Appendix 1. PRISMA 2009 Checklist. Appendix 2. Search strategy. Supplementary Figure 1. The Egger’s test of mortality. Supplementary Figure 2. Sensitivity analysis of length of stay. Supplementary Figure 3. Pooled effect estimates for the impact on length of stay after removing the study by Selby [18]. Supplementary Figure 4. The Egger’s test of length of stay. Supplementary Figure 5. Sensitivity analysis for AKI progression. Supplementary Figure 6. Pooled effect estimates for the impact on AKI progression after removing the study by Kolhe [22]. Supplementary Figure 7. The Egger’s test of AKI-RRT usage. Supplementary Figure 8. The Egger’s test of AKI progression. Supplementary Table 1. Summary of clinical outcome for studies using only e-alert system. Supplementary Table 2. Studies with a design of care bundles only. [file 12882_2021_2459_MOESM1_ESM.docx]

**Supplementary materials**

**Appendix 1**

PRISMA 2009 Checklist

| Section/topic | # | Checklist item | Reported on page # |
| --- | --- | --- | --- |
| TITLE | | |  |
| Title | 1 | Identify the report as a systematic review, meta-analysis, or both. | 1 |
| ABSTRACT | | |  |
| Structured summary | 2 | Provide a structured summary including, as applicable: background; objectives; data sources; study eligibility criteria, participants, and interventions; study appraisal and synthesis methods; results; limitations; conclusions and implications of key findings; systematic review registration number. | 1 |
| INTRODUCTION | | |  |
| Rationale | 3 | Describe the rationale for the review in the context of what is already known. | 1 |
| Objectives | 4 | Provide an explicit statement of questions being addressed with reference to participants, interventions, comparisons, outcomes, and study design (PICOS). | 2 |
| METHODS | | |  |
| Protocol and registration | 5 | Indicate if a review protocol exists, if and where it can be accessed (e.g., Web address), and, if available, provide registration information including registration number. | 2 |
| Eligibility criteria | 6 | Specify study characteristics (e.g., PICOS, length of follow-up) and report characteristics (e.g., years considered, language, publication status) used as criteria for eligibility, giving rationale. | 2 |
| Information sources | 7 | Describe all information sources (e.g., databases with dates of coverage, contact with study authors to identify additional studies) in the search and date last searched. | 2, Supplementary Appendix 2 |
| Search | 8 | Present full electronic search strategy for at least one database, including any limits used, such that it could be repeated. | Supplementary Appendix 2 |
| Study selection | 9 | State the process for selecting studies (i.e., screening, eligibility, included in systematic review, and, if applicable, included in the meta-analysis). | 2 |
| Data collection process | 10 | Describe method of data extraction from reports (e.g., piloted forms, independently, in duplicate) and any processes for obtaining and confirming data from investigators. | 2 |
| Data items | 11 | List and define all variables for which data were sought (e.g., PICOS, funding sources) and any assumptions and simplifications made. | 2 |
| Risk of bias in individual studies | 12 | Describe methods used for assessing risk of bias of individual studies (including specification of whether this was done at the study or outcome level), and how this information is to be used in any data synthesis. | 2 |
| Summary measures | 13 | State the principal summary measures (e.g., risk ratio, difference in means). | 2 |
| Synthesis of results | 14 | Describe the methods of handling data and combining results of studies, if done, including measures of consistency (e.g., I^2^) for each meta-analysis. | 2 |
| Risk of bias across studies | 15 | Specify any assessment of risk of bias that may affect the cumulative evidence (e.g., publication bias, selective reporting within studies). | 2 |
| Additional analyses | 16 | Describe methods of additional analyses (e.g., sensitivity or subgroup analyses, meta-regression), if done, indicating which were pre-specified. | 2 |
| RESULTS | | |  |
| Study selection | 17 | Give numbers of studies screened, assessed for eligibility, and included in the review, with reasons for exclusions at each stage, ideally with a flow diagram. | 3 |
| Study characteristics | 18 | For each study, present characteristics for which data were extracted (e.g., study size, PICOS, follow-up period) and provide the citations. | 3 |
| Risk of bias within studies | 19 | Present data on risk of bias of each study and, if available, any outcome level assessment (see item 12). | 3, Figure 2 |
| Results of individual studies | 20 | For all outcomes considered (benefits or harms), present, for each study: (a) simple summary data for each intervention group (b) effect estimates and confidence intervals, ideally with a forest plot. | 3 |
| Synthesis of results | 21 | Present results of each meta-analysis done, including confidence intervals and measures of consistency. | 3-7 |
| Risk of bias across studies | 22 | Present results of any assessment of risk of bias across studies (see Item 15). | 3, Figure 2 |
| Additional analysis | 23 | Give results of additional analyses, if done (e.g., sensitivity or subgroup analyses, meta-regression [see Item 16]). | 4-7 |
| DISCUSSION | | |  |
| Summary of evidence | 24 | Summarize the main findings including the strength of evidence for each main outcome; consider their relevance to key groups (e.g., healthcare providers, users, and policy makers). | 7 |
| Limitations | 25 | Discuss limitations at study and outcome level (e.g., risk of bias), and at review-level (e.g., incomplete retrieval of identified research, reporting bias). | 9 |
| Conclusions | 26 | Provide a general interpretation of the results in the context of other evidence, and implications for future research. | 9 |
| FUNDING | | |  |
| Funding | 27 | Describe sources of funding for the systematic review and other support (e.g., supply of data); role of funders for the systematic review. | 9 |

**Appendix 2**

Search strategy

2019.5.23 pubmed

(((【(Acute kidney injury)[Title/Abstract] OR AKI[Title/Abstract] OR (Acute kidney disease)[Title/Abstract] OR akd[Title/Abstract] OR “Hepatorenal syndrome”[Title/Abstract] OR “acute Glomerulonephritis”[Title/Abstract] OR (rapidly progressive Glomerulonephritis)[Title/Abstract] OR Nephrotoxicity[Title/Abstract] OR (Acute[Title/Abstract] AND (renal[Title/Abstract] OR kidney)[Title/Abstract] AND (injury[Title/Abstract] OR failure[Title/Abstract] OR insufficiency[Title/Abstract] OR damage[Title/Abstract] OR dysfunction[Title/Abstract] OR impairment))】[Title/Abstract])) AND (【(Electronic alert)[Title/Abstract] OR E-alert[Title/Abstract] OR (E alert)[Title/Abstract] OR (Laboratory Automation)[Title/Abstract] OR (alarm[Title/Abstract] OR alert[Title/Abstract] OR “information system”[Title/Abstract] OR reporting system[Title/Abstract] OR warn)[Title/Abstract] OR (Alarm monitor)[Title/Abstract] OR (Clinical Alarms)[Title/Abstract] OR (Physiologic Monitoring)[Title/Abstract] OR (Point-of-Care Systems)[Title/Abstract] OR “Reminder Systems”[Title/Abstract] OR “Medication alert system”[Title/Abstract] OR (Automated surveillance)[Title/Abstract] OR (Surveillance system)】[Title/Abstract]))) OR (((【(Acute kidney injury)[Title/Abstract] OR AKI[Title/Abstract] OR (Acute kidney disease)[Title/Abstract] OR akd[Title/Abstract] OR “Hepatorenal syndrome”[Title/Abstract] OR “acute Glomerulonephritis”[Title/Abstract] OR (rapidly progressive Glomerulonephritis)[Title/Abstract] OR Nephrotoxicity[Title/Abstract] OR (Acute[Title/Abstract] AND (renal[Title/Abstract] OR kidney)[Title/Abstract] AND (injury[Title/Abstract] OR failure[Title/Abstract] OR insufficiency[Title/Abstract] OR damage[Title/Abstract] OR dysfunction[Title/Abstract] OR impairment))】[Title/Abstract])) OR ([(Care bundle*) or (healthcare bundle) or (patient care bundle) or (patient care bundles) or (Decision support system) or (clinical decision support system*) or (Computer-Assisted Drug Therapy) or (Computerized decision support) or (Computerized order entry system) or (Computerized and (physician or provider) and (order entry system)) or CPOE or (Electronic order entry system) or (Electronic Health Record) or (Health Information Systems) or (hospital lnformation Systems) or (Medical Order Entry) or (Computerized Medical Records Systems) or (Hospital Medication Systems)])

【(Acute kidney injury) or AKI or (Acute kidney disease) or akd or “Hepatorenal syndrome” or “acute Glomerulonephritis” or (rapidly progressive Glomerulonephritis) or Nephrotoxicity or (Acute and (renal or kidney) and (injury or failure or insufficiency or damage or dysfunction or impairment))】

2019.5.23 the cochrane

(【(Acute kidney injury) or AKI or (Acute kidney disease) or akd or “Hepatorenal syndrome” or “acute Glomerulonephritis” or (rapidly progressive Glomerulonephritis) or Nephrotoxicity or (Acute and (renal or kidney) and (injury or failure or insufficiency or damage or dysfunction or impairment))】AND【((Electronic alert) or E-alert or (E alert) or (Laboratory Automation) or (alarm or alert or “information system” or reporting system or warn) or (Alarm monitor) or (Clinical Alarms) or (Physiologic Monitoring) or (Point-of-Care Systems) or “Reminder Systems” or “Medication alert system” or (Automated surveillance) or (Surveillance system))or ((Care bundle*) or (healthcare bundle) or (patient care bundle) or (patient care bundles) or (Decision support system) or (clinical decision support system*) or (Computer-Assisted Drug Therapy) or (Computerized decision support) or (Computerized order entry system) or (Computerized and (physician or provider) and (order entry system)) or CPOE or (Electronic order entry system) or (Electronic Health Record) or (Health Information Systems) or (hospital lnformation Systems) or (Medical Order Entry) or (Computerized Medical Records Systems) or (Hospital Medication Systems))】

(Care bundle*) or (healthcare bundle) or (patient care bundle) or (patient care bundles) or (Decision support system) or (clinical decision support system*) or (Computer-Assisted Drug Therapy) or (Computerized decision support) or (Computerized order entry system) or (Computerized and (physician or provider) and (order entry system)) or CPOE or (Electronic order entry system) or (Electronic Health Record) or (Health Information Systems) or (hospital lnformation Systems) or (Medical Order Entry) or (Computerized Medical Records Systems) or (Hospital Medication Systems)

2019.5.23 Embase

#1 ('acute kidney failure'/exp OR 'acute kidney tubule necrosis'/exp) AND ('electronic alert'/exp OR 'electronic alert system'/exp OR 'e alert' OR 'monitor'/exp OR 'biomedical monitors' OR 'monitor' OR 'monitor, device (physical object)' OR 'monitors' OR 'alarm monitor'/exp OR 'alarm monitor' OR 'clinical alarms' OR 'physiologic monitoring'/exp OR 'monitoring, physiologic' OR 'physiologic monitoring' OR 'point of care system'/exp OR 'reminder system'/exp OR 'medication alert system' OR 'automated surveillance' OR 'surveillance'/exp OR 'smartphone'/exp OR 'smart phone' OR 'smartphone' OR 'smartphones')

#2 ('acute kidney failure'/exp OR 'acute kidney tubule necrosis'/exp OR 'kidney disease'/exp) AND ('care bundles' OR 'care bundle'/exp OR 'decision support system'/exp OR 'clinical decision support system'/exp OR 'computer assisted drug therapy'/exp OR 'computerized decision support system'/exp OR 'computerized order entry system' OR 'order entry system' OR 'electronic health record'/exp OR 'electronic health record' OR 'electronic health records' OR 'medical information system'/exp OR 'physician order entry system'/exp OR 'hospital information system'/exp)

**Supplementary Figure 1. The Egger’s test of mortality.**

**Supplementary Figure 2. Sensitivity analysis of length of stay.**

**
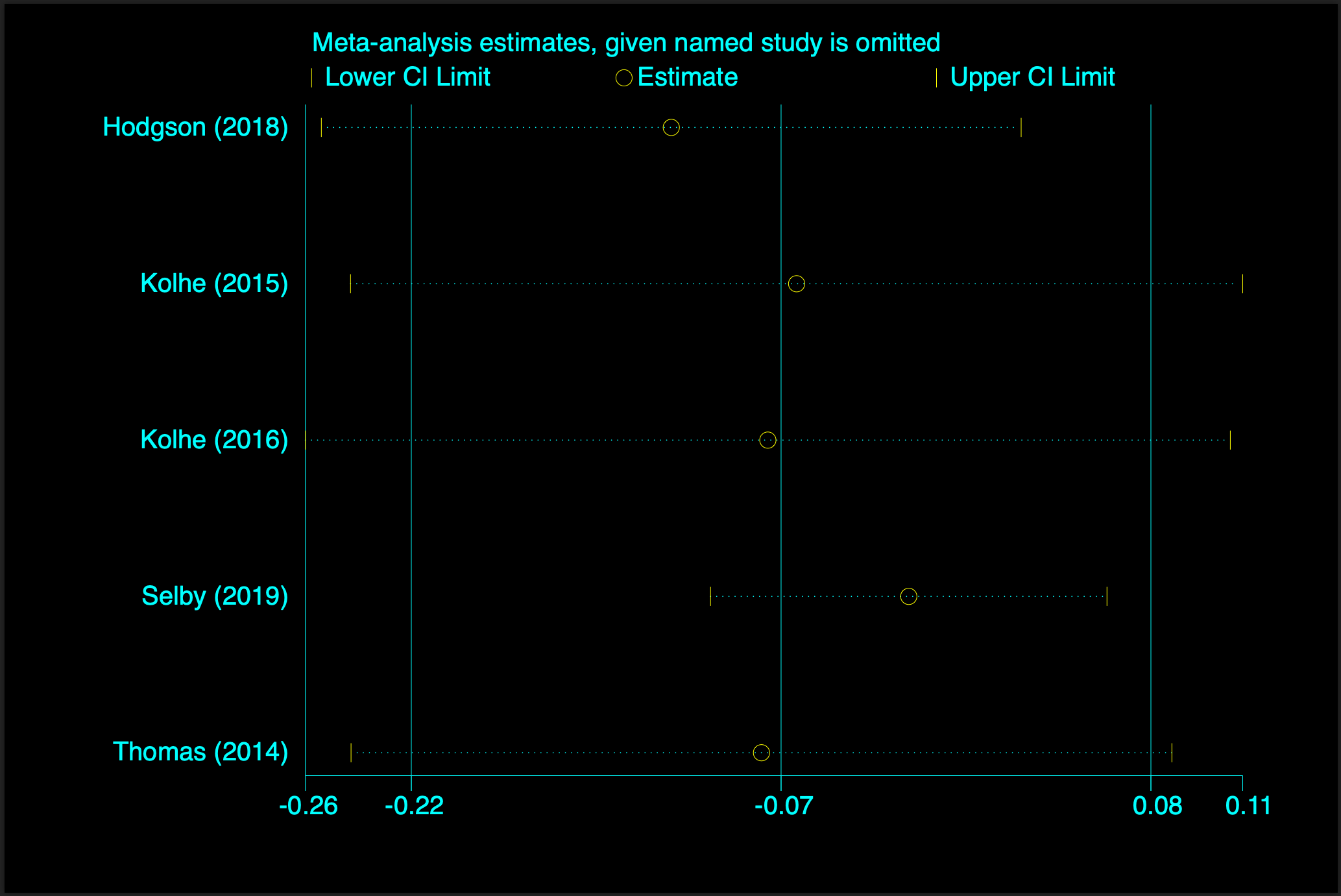
**

**Supplementary Figure 3. Pooled effect estimates for the impact on length of stay after removing the study by Selby.**

**Supplementary Figure 4. The Egger’s test of length of stay**

**Supplementary Figure 5. Sensitivity analysis for AKI progression**

**
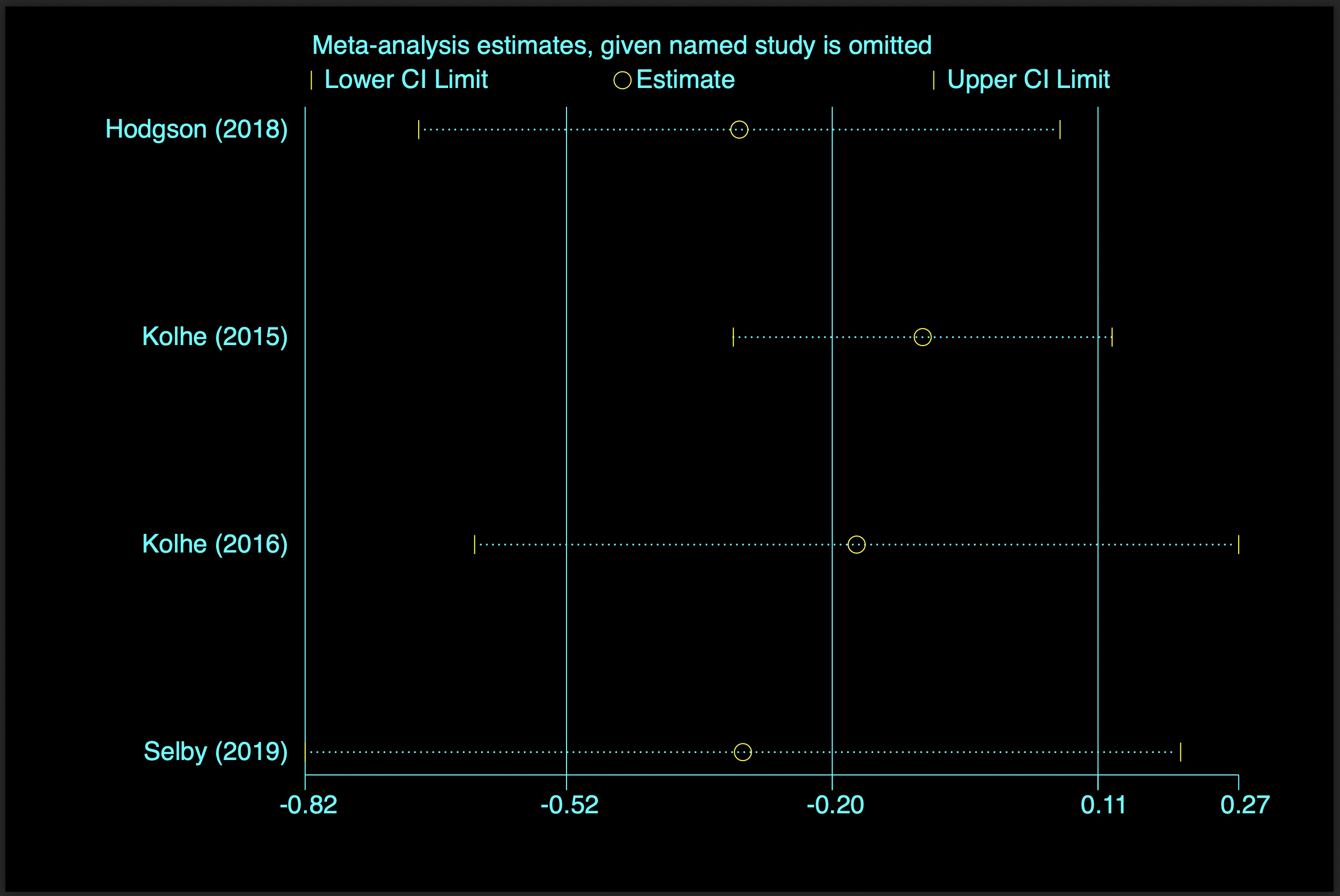
**

**Supplementary Figure 6. Pooled effect estimates for the impact on AKI progression after removing the study by Kolhe [22]**

**Supplementary Figure 7. The Egger’s test of AKI-RRT usage**

**Supplementary Figure 8. The Egger’s test of AKI progression**

**Supplementary Table 1. Summary of clinical outcome for studies using only e-alert system**

| Study | Journal | Design | Context | | Number of patients | Outcomes | | |
| --- | --- | --- | --- | --- | --- | --- | --- | --- |
|  |  |  | Country | Setting |  |  |  |  |
|  |  |  |  |  |  | Patient-ralated outcome | Process of care outcome | Health service use outcome |
| Rind 1994 [11] | Arch Intern Med | Time series | USA | Mix | 922 | No difference in mortality, renal function, worsening AKI | Decreased Time to medication adjustment | No difference in length of stay, total cost |
| Colpaert 2012 [12] | Crit Care Med | Time series | Belgium | ICU | 951 | No difference in mortality, frequency of RRT and maximum AKI stage | Increased management including fluid therapy, diuretics or vasopressors | No difference in ICU length of stay |
| Selby 2012 [26] | CJASN | Time series | UK | Mix | 4159 | Decreased mortality; no differences in renal recovery | NA | No differences in length of stay |
| Wilson 2015 [9] | Lancet | Randomized clinical trial | USA | Mix | 2393 | No difference | No difference | No difference |
| Park 2018 [27] | AJKD | Before-after | Korea | Mix | 3193 | Decreased rate of severe AKI, increased rate of AKI recovery | Decreased overlooked AKI incident cases | NA |
| Al-jaghbeer 2018 [28] | JASN | Before-after | USA | Mix | 64512 | Decreased crude mortality and need for dialysis rate | Decreased Days of receiving nephrotoxic medications | Decreased length of stay |
| Kothari 2018 [29] | Acad Pathol | Time-series | USA | Mix | 23460 | NA | Increased Documentation | NA |
| Tollitt 2018 [30] | Family Practice | 2x2 factorial design project | UK | Primary care | 1260 | Decreased mortality | Decreased response time to AKI 2 and 3 | NA |
| Wu 2018 [31] | International Urology and Nephrology | Randomized clinical trial | China | ICU | 875 | No difference in mortality, dialysis, rehabilitation of renal function | Increased nephrology consult | NA |
| Aiyegbusi 2019 [32] | Clinical Kidney Journal | Before-after | UK | Primary care | 3462 | Increased rates of hospitalization | Increased rates of creatinine monitoring | NA |
| Collaborative 2019 [33] | Clinical Medicine | Before-after | UK | Acute medicine | 613 | NA | Increased recognition and management | NA |
| Pou 2019 [34] | European Journal of Hospital Pharmacy | Before-after | Spain | Mix | 2006 | NA | Increased incidence of changes or discontinuation of the nephrotoxic drug | NA |

Note: Abbreviations: NA, not available; ICU, intensive care unit.

**Supplementary Table 2. Studies with a design of care bundles only**

| Study | Setting | Study design | Care bundles | Outcomes |
| --- | --- | --- | --- | --- |
| Ford 2012 [35] | Surgical wards and medical admission unit | Sample size not reported; Before-after | Five elements: medication review; manage hypotension; fluid balance; urinalysis; exclude obstruction | In the post-implementation phase, 100% AKI recognition, 80% care bundle completion in 67% of AKI cases. No process measures or patient outcomes were reported. |
| Tsui 2014 [36] | Medical admission unit | 100 patients, before-after | Record baseline creatinine, assess fluid status, urinalysis, medication review, urine protein/creatinine ratio; monitor urine output; renal US; referral elements | Improvements in process of care and patient-related outcomes |
| Joslin 2015 [37] | Hospital wide | 192 patients, before-after | Patient assessment; fluid therapy; manage hyperkalaemia; urinalysis; medication review; repeat serum creatinine; renal US; fluid balance charting | Improvements in process of care. |

Note: Abbreviations: AKI, acute kidney injury.
